# Resilience Improves the Sleep Quality in Disabled Elders: The Role of Perceived Stress

**DOI:** 10.3389/fpsyg.2021.585816

**Published:** 2021-02-11

**Authors:** Yumei Cai, Junlei Wang, Liwen Hou

**Affiliations:** ^1^School of Social and Public Administration, East China University of Science and Technology, Shanghai, China; ^2^Law School, East China University of Science and Technology, Shanghai, China

**Keywords:** resilience, sleep quality, perceived stress, disabled elders, nursing house

## Abstract

The current research aims to prove the impact of resilience on sleep quality and to investigate the mediating function of perceived stress in the paths from resilience to sleep quality among disabled Chinese elders. The participants were 196 elders with visual and physical disability who resided in the nursing houses in Shanghai, including 102 males and 94 females whose mean age was 74.5 years old (standard deviation = 6.81). All the elders were measured with the 10-item Connor-Davidson Resilience Scale, Pittsburgh Sleep Quality Index (PSQI), and Perceived Stress Scale. The results showed that all the demographic variables were significantly related to perceived stress and PSQI scores. High levels of resilience could significantly enhance the quality of sleep in disabled Chinese elders. In addition, the two-step mediation models also confirmed the impact of resilience on sleep quality as mediated through perceived stress in this special aged population. Better knowledge on the mechanisms of sleep quality among older adults could benefit the prevention and treatment of some geriatric diseases.

## Introduction

Sleep quality is one of the most important aspects for keeping fine physical health status in older adults. Together with exercise and diet, sleep has been identified as a key lifestyle factor to maintain the subjective psychological well-being among elder population (Watanabe et al., 2020). Surveys have indicated that poor quality of sleep was a strong risk factor for psychiatric disorders (Baglioni et al., 2011; Cable et al., 2017), such as anxiety and depression, and even dementia and cognitive impairment (Sexton et al., 2017; Suh et al., 2018; Cordone et al., 2019; Cordone and De Gennaro, 2020) in the older population. Therefore, having better knowledge on the mechanisms and related factors of sleep quality among older adults could benefit the prevention and treatment of some geriatric diseases.

Population aging has become a major challenge over the whole world (Wang et al., 2018). Along with the 21st century, human beings have gradually stepped into an aging society. According to the World Population Prospects 2019, people aged 65 years and older accounted for 1/11 of the world’s total population and was predicted to increase to 1/6 in 2050 [United Nations (UN), 2019]. China is one of the countries with the largest number of senior citizens in the world. According to the statistics, there are totally 212 million older adults aged 60 years and older in China in 2014 [National Bureau of Statistics of China (NBSC), 2015], and by 2030, the number may rise to a higher level of 358 million [United Nations (UN), 2015]. Such a huge population could bring great dangers and has aroused widespread concern in the whole society. Sleep quality is one of the most important focus for the Chinese elders. More than 1/3 of the elder population in China had difficulties in sleeping, such as insomnia and poor quality (Wang et al., 2020). Previous studies have shown that sleep quality is related to health status in elders. Poor quality of sleep not only seriously affects the quality of life (Wang F. et al., 2019) but also leads to serious psychosomatic problems (e.g., loneliness and cognitive impairment) among Chinese elders (Suh et al., 2018; Yu et al., 2018). Long-term insomnia would lead to a decline in body function as well as an increase in the risk of various mental disorders, physical illnesses, and mortality (Bazargan et al., 2019). However, most of the current research made concerns on the general elders. There is little focus for the special older population, such as in terms of disability, empty nest, and mobility. Disabled elder refers to the older adult who suffered from the long-term impairment of body, language, hearing, spirit, intelligence, or multiple aspects. At the end of 2016, approximately 18.3% of Chinese elders were disabled and half-disabled, which reached a large number of 40.63 million [China National Committee on Aging (CNCA), 2016]. These disabled older adults endure the dual destruction of both spirit and body, as well as accompanying insomnia (Tan et al., 2019), but attention was paid to these older population. The existing research would like to explore the mechanisms of sleep quality among this special population—disabled Chinese elders who lived in nursing houses.

For the intelligent old-age model and healthy life management of disabled elders, advocating prevention and active response is usually used in the perspective of active pension (Godfrey and Johnson, 2009; Zhang and Han, 2017). Resilience is one of the most commonly mentioned variables in the perspective of active pension. Resilience is defined as the capacity to successfully adapt and overcome difficulties or challenges in life and to experience processes and outcomes, especially high-stress or traumatic events (Halilova et al., 2020). When suffering from stress and trauma, resilience was considered as an important factor in maintaining human’s psychological well-being (Wang L. et al., 2019). Resilience is usually divided into two types. One is an explanatory ability or trait: a stable personality trait or ability that can overcome risk factors and avoid damage to developmental function, adaptive ability, mental health, etc. (Zeng and Li, 2003). The other one is considered as a positive psychological outcome or adaptation process (Yao, 2012). Evidence from previous research has indicated that both of the two forms of psychological resilience positively predicted sleep quality. As an ability or trait, sleep disorders could be reduced by resilience-improving training in junior athletes (Hrozanova et al., 2019). As a positive psychological outcome or adaptation process, individuals could decrease sleep disorders by adjusting and adapting this prosses among left-behind children (Yao, 2012). By sampling the older women who resided in communities, the cross-sectional investigation conducted in New York revealed that resilience had emerged as a positive and strong independent predictor of sleep quality (Blanc et al., 2020). Similarly, the longitudinal survey with a great sample of pregnant women in Finland demonstrated that a higher level of resilience predicted better sleep quality (Der Zwan et al., 2017). Meanwhile, this research also found that people with poor mental health (e.g., anxiety) should also be screened for sleep disorders. Thus, resilience is a positive predictor of sleep quality. A strong level of psychological resilience helps to improve the quality of sleep.

Except resilience, there are also many other variables that linked to one’s sleep quality, including loneliness, anxiety, and physical health. Among these variables, perceived stress is identified as one of the interesting factors for sleep quality. Perceived stress is defined as the subjective perception of an individual’s internal and external stress events, which can alter a person’s cognitive function and affect his or her emotional and physiological status (Vaneck et al., 1996). Perceived stress has been reported to be strongly and negatively related to sleep quality. An increasing body of literature indicated that increased perceived stress would make sleep quality poorer (Liu et al., 2017; Lamis et al., 2018; Smith-Mason and Gil-Rivas, 2019). Perceived stress causes physical and mental reaction, and these physical and mental adjustments form the basis of changes in sleep quality (Yan et al., 2010). Investigating 1,032 elder adults in Liaoning Province, Liu et al. (2017) revealed that all dimensions of sleep quality were negatively associated with perceived stress. In addition, they further confirmed the mediating function of sleep quality in the path from stress to depression. The negative relationship between stress cognitive processes and sleep quality can be understood by referring to the insomnia cognitive model. The excessive and uncontrollable concerns about life stress before going to bed causes emotional arousal, which would lead to cognitive bias in life stress events and increase the concern about the threat of stress. Therefore, people had a distorted evaluation of the stressful event and had a subjective perception of decline in sleep quality. What is more, a great deal of survey have also evaluated the links from resilience to perceived stress and indicated a negative correlation between these two variables (Sarrionandia et al., 2018; Wang L. et al., 2019). By recruiting 138 participants with heroin addiction at Nanjing Shifosi Addiction Rehabilitation Center, Wang L. et al. (2019) confirmed that resilience negatively predicted perceived stress and depression as well as played mediating roles in the association between the latter two factors. Thus, from the discussion above, we can know that resilience, sleep quality, and perceived stress were pairwise correlated. A high level of resilience could improve the quality of sleep. Perceived stress has also been confirmed to negatively predict resilience and sleep quality. In addition, previous investigation also demonstrated the mediating function of stress in the path from psychological resilience to some behavioral variables (e.g., adverse childhood experience; Sun and Singer, 2018). As one of the important impact factors of sleep quality, perceived stress may also play the same role with the relation of resilience and sleep quality. However, related research is still insufficient.

In general, the current research aims to prove the impact of resilience on sleep quality and to investigate the mediating function of perceived stress in the paths from resilience to sleep quality among the disabled elders who resided in nursing house in China. We will mainly explore two hypotheses. One is that resilience significantly improves the sleep quality of disabled Chinese elders. In addition, the path from resilience to sleep quality is mediated through perceived stress.

## Materials and Methods

### Samples and Procedures

The data collected were from the research conducted which was a cross-sectional investigation, which evaluated the psychical and psychological health status among disabled elders. All the disabled elders were recruited from eight public nursing houses located in Shanghai, the biggest economic center in China. A convenience sampling method was employed to select the participants. The participants’ inclusion criteria were as follows: (1) age of more than 60 years old, (2) disabled (e.g., visual and physical disability), (3) able to communicate, and (4) voluntary participation in the survey and signed the written informed consent form. Finally, 246 eligible elders were invited to participate in the investigation. Well-trained researchers read the self-reported scales to the disabled elders one by one and face to face to ensure the accuracy of the investigation. Then, 196 valid questionnaires were returned, indicating a validity rate of 79.67%. The research was approved by the ethics committee of East China University of Science and Technology (ECUST No. 20181130b0601231), and all procedures followed the Declaration of Helsinki.

### Measurement Tools

#### Demographic Variables

We employed age, gender, ethnicity, marital status, education status, and disability type to measure each disabled elder’s demographic characteristics. Among these control variables, gender was coded as 0 = “male” and 1 = “female.” The participants’ marital status was assessed using the question “Have you been married?”, with the answer choices of 0 = “married” or 1 = “unmarried.” Similarly, education status was assessed using the question “Have you been educated?,” with the answer 0 = “educated” or 1 = “uneducated.”

#### Resilience

We administered the Chinese version of the 10-item Connor–Davidson Resilience Scale (CD-RISC-10; Connor and Davidson, 2003) to assess each disabled elder’s mental resilience, which referred to a five-point Likert form ranging from 0 = “never” to 4 = “always.” The sum of each item makes up the total score, in the range of 0 to 40. A higher score shows lower levels of psychological resilience problems. Previous investigation has revealed the CD-RISC satisfactory internal consistency and validity among the elders in China (Wu et al., 2018). The internal consistency for the existing investigation was 0.76.

#### Sleep Quality

We employed the Chinese version of the Pittsburgh Sleep Quality Index (PSQI; Buysse et al., 1989) to measure each disabled elder’s sleep quality. Seven dimensions were included in the questionnaire, which referred to a four-point Likert form, ranging from 0 to 4. The sum of each item makes up the total score, in the range of 0–21. A higher score shows more severe sleep disorders. A previous survey has revealed the PSQI to have satisfactory internal consistency and validity among Chinese elders (Qian et al., 2017). The internal consistency for the existing investigation was 0.77.

#### Perceived Stress

We administered the Chinese version of the Perceived Stress Scale (PSS; Cohen et al., 1983) to assess each disabled elder’s perceived stress. Fourteen self-reported items were included in the questionnaire, which referred to a five-point Likert form, ranging from 0 = “never” to 4 = “very often.” The sum of each item makes up the total score, in the range of 0–56. A higher score shows more severe stress. A previous research has demonstrated the PSS to have satisfactory internal consistency and validity among Chinese elders (Wu et al., 2018). The internal consistency for the existing investigation was 0.86.

### Statistical Analysis

SPSS 23.0 for Windows was employed to analyze the data. Cronbach’s alpha coefficient was conducted to assess the reliability of the questionnaires. We performed descriptive statistics to provide information about the demographic characteristics of the disabled elders. Pearson Correlation analysis was used to conduct the correlation matrix among demographic variables, resilience, perceived stress, and sleep quality in disabled elders. SPSS 23.0 and PROCESS program for SPSS were adopted to evaluate the mediating effects of the relationships among resilience, perceived stress, and sleep quality (Hayes, 2012). As the hypothesis model showed, resilience referred to the independent variable. Perceived stress was identified as the mediating variable. Sleep quality acted as the outcome variable. The two-step method described by Baron and Kenny (1986) was adopted to evaluate both the direct and indirect impacts of perceived stress on sleep quality. Firstly, the hierarchical regression model of sleep quality was used to preliminarily calculate the mediating role. Then, the bootstrapping method defined by Preacher and Hayes (2008) was adopted to further test the indirect impacts of resilience on sleep quality. The assessment sampled 5,000 times and calculated the 95% confidence intervals (CI). If 0 did not involve the range of 95% CI, the indirect link was considered as significant. We considered *P* < 0.05 (two-tailed) as significant for the existing research.

## Results

### Characteristics

There are totally 196 elders living in a nursing house who participated in the survey, including 185 physically disabled and 11 visually disabled elders. Their average age was 74.5 years, with the standard deviation of 6.81. Most of these disabled elders were male (*n* = 102, 52.04%), Han (*n* = 172, 87.76%), unmarried (*n* = 112, 57.14%), and illiterate (*n* = 135, 68.88%). In general, the mean score of the sleep quality for the disabled elders was 14.24, with the standard deviation of 6.85. The mean scores of resilience and perceived stress were 27.20 (standard deviation = 10.01) and 33.58 (standard deviation = 14.26), respectively. Table 1 provides more detailed information on the characteristics of the disabled elders.

**TABLE 1:** Characteristics and sleep quality in disabled elders (*N* = 196).

Demographics	*N* (%)/mean ± SD
Age	74.53 ± 6.81
**Gender**	
0 = “Male”	94 (47.96)
1 = “Female”	102 (52.04)
**Ethnicity**	
0 = “Han”	172 (87.76)
1 = “Others”	24 (12.24)
**Marital status**	
0 = “Married”	84 (42.86)
1 = “Unmarried”	112 (57.14)
**Education**	
0 = “Educated”	41 (20.92)
1 = “Uneducated”	155 (79.08)
**Disability type**	
0 = “Visual”	11 (5.6)
1 = “Physical”	185 (94.4)
Resilience	27.20 ± 10.01
Perceived stress	33.58 ± 14.26
Sleep quality	14.24 ± 6.85

### Correlation

We conducted a Pearson Correlation analysis for the demographic variables and variables of interest. The results showed that all demographic variables were significantly linked to perceived stress and PSQI scores. Age was negatively associated with resilience (*r* = −0.208, *P* < 0.01), while education status had a positive influence on resilience (*r* = 0.166, *P* < 0.05). In addition, bivariate correlations also showed resilience to be negatively related to the scores of PSQI (*r* = −0.303, *P* < 0.001) and perceived stress (*r* = −0.386, *P* < 0.001). Perceived stress was positively related to the scores of PSQI (*r* = 0.441, *P* < 0.001). Table 2 provides detailed information on the correlation analysis.

**TABLE 2:** Correlation analysis of the involved variables.

Variables	1	2	3	4	5	6	7
Age	–						
Gender	0.084	–					
Marital status	–0.108	0.045	–				
Education	0.102	−0.198**	–0.061	–			
Resilience	−0.208**	–0.112	–0.105	0.166*	–		
Perceived stress	0.261***	0.159*	0.163*	−0.205**	−0.303***	–	
Pittsburgh Sleep Quality Index	0.246***	0.201**	0.193**	−0.246***	−0.386***	0.441***	–

*P < 0.05 (two-tailed); **P < 0.01 (two-tailed); and ***P < 0.001 (two-tailed).

### Indirect Link From Resilience to Sleep Quality

We adopted two steps, including the hierarchical regression model and bootstrapping sampling, to test the indirect impact of resilience on sleep quality *via* perceived stress. Table 3 provides detailed information on the hierarchical linear regressions. The results showed that, after controlling the demographic variables, resilience was negatively associated with PSQI scores (β = −0.322, *P* < 0.001). Then, we assessed the mediating effects of perceived stress. After adding perceived stress, the link from resilience to PSQI scores was still significant (β = −0.194, *P* < 0.001). In addition, when inserting perceived stress into the impact of resilience on the PSQI scores, the absolute value of β significantly cut down from 0.322 to 0.194, which preliminarily demonstrated the mediating function of perceived stress.

**TABLE 3:** Hierarchical regression model of Pittsburgh Sleep Quality Index.

	Step 1		Step 2		Step 3	
	*B*	β	*B*	β	*B*	β
Age	0.203	0.203	0.168	0.168	0.045	0.045
Gender	0.112	0.059	0.094	0.055	0.052	0.023
Marital status	0.234	0.112	0.186	0.124	0.043	0.018
Education	–0.355	–0.120	–0.278	–0.176	–0.135	–0.061
Resilience			–0.322	–0.322	–0.194	–0.194
Perceived stress					0.411	0.411
*R*^2^	0.395		0.502		0.646	
Δ*R*^2^	0.395*		0.107*		0.144*	

B, unstandardized coefficient; β, standardized coefficient. *P < 0.001 (two-tailed).

**FIGURE 1:**
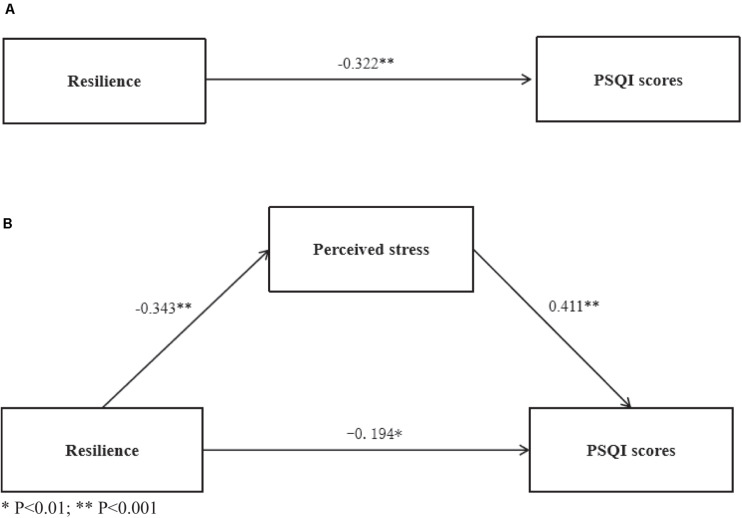
The mediation models among resilience, perceived stress, and Pittsburgh Sleep Quality Index scores. ^∗^*P* < 0.01; ^∗∗^*P* < 0.001.

With the program PROCESS, we determined the bootstrap sampling method. Table 4 and Figure 1 provide detailed information. The results indicated a negative influence of resilience on PSQI scores [coefficient = −0.322, 95% CI = (−0.445, −0.174)], and this negative relationship still remained strong after adding the perceived stress [coefficient = −0.194, 95% CI = (−0.301, −0.045)]. In addition, 0 was not involved in the 95% CI, which further confirmed the hypothesis that perceived stress mediated the links from resilience to sleep quality.

**TABLE 4:** Direct and indirect effect analysis (bootstrap estimation).

Pathway	Coefficient	SE	95% CI	
			Lower	Upper
**Direct effects**				
Resilience–Pittsburgh Sleep Quality Index (PSQI)	−0.322	0.077	−0.445	−0.174
**Indirect effects**				
Resilience–perceived stress–PSQI	−0.194	0.041	−0.301	−0.045

## Discussion

To the best of our knowledge, our research is the first study to explore the relationship among resilience, sleep quality, and perceived stress for a special older population—disabled elders in China—as well as the first attempt to test the role of perceived stress in the path from resilience to sleep quality. We found that all the demographic variables were significantly related to perceived stress and PSQI scores. In line with our hypothesis, resilience was negatively linked to the PSQI scores, meaning that high levels of resilience could significantly enhance the quality of sleep in the disabled Chinese elders. In addition, the two-step mediation models also confirmed that the impact of resilience on sleep quality was mediated through perceived stress in this special aged population.

For the correlation section, we used Pearson correlation analysis to test the bivariate correlation among demographic factors and targeted variables. Our research showed that education levels were positively correlated with sleep quality, while age, gender, and marital status had negative impacts on sleep quality. These finding were consistent with previous research (Bote et al., 2006; Hrozanova et al., 2019). For instance, in the investigation conducted by Hrozanova et al. (2019), the female gender accounted for approximately 2% of the variance for poor quality of sleep, which may be caused by the general experience of worry and stress of women (Matud, 2004; Hrozanova et al., 2019). In addition, we also found that resilience was strongly and negatively linked to the PSQI scores and perceived stress, that is, it could effectively improve the sleep quality and reduce the levels of perceived stress by improving psychological resilience. Several surveys conducted before supported our findings (Lamis et al., 2018; Li et al., 2019; Blanc et al., 2020). Using a big sample of college students (*n* = 1065), Li and his colleagues indicated the direct effect of resilience on sleep quality. The negative predicted function of perceived stress on sleep quality has also been checked and confirmed among the elderly (Liu et al., 2017).

In the indirect association section, we evaluated whether perceived stress played the buffer role in the influence of resilience on sleep quality or not. Our findings illuminated that perceived stress acted as the mediator that accounted for the link from resilience to sleep quality, which is in line with our hypothesis. The hyperarousal theory for insomnia described by Morin et al. (2003) has indicated that sleep disturbance may be strongly affected by cognitive arousal (Morin et al., 2003). Persons who obtained a higher level of perceived stress were more inclined to own negative reappraisals for the past, which would raise their cognitive arousal and conversely lead to poorer quality of sleep.

Several limitations of this research should also be acknowledged. Firstly, the study was designed as a cross-sectional form, which could not affirm the causal associations between resilience, sleep quality, and perceived stress. The research firstly explored the links from resilience to sleep quality through evaluating the role of perceived stress in disabled elders. However, these hypothesis associations were limited by theory and survey. Longitudinal studies were needed to confirm the paths. Secondly, we conducted convincing sampling methods to select the participants. Methodological limitations may be caused by the non-random selection. Thirdly, all the questionnaires, like PSQI, used in the investigation were self-reported, have subjectivities to some extent, and may lead to measure bias. Finally, we collected disabled elders from eight nursing houses in Shanghai, which is probably the most developed city in China. Thus, it limited the generalization of our findings to disabled elders in other areas. More diversified research should be conducted in the future.

Apart from these limitations, the existing research highlight several implications in both theory and reality. Based on the special older population—disabled Chinese elders who resided in a nursing house—our data offered new evidence to further confirm the strong prediction of resilience on sleep quality. Sleep disorder was one of the most common hazards to elders, which would raise the risk for physical and psychological diseases (Luo et al., 2013). In addition to medication, no adequate effective physical therapy was available to solve sleep disorders. Based on the findings of the present research, effective exercise and training of psychological resilience may play important roles in significantly improving the quality of sleep among older adults. In addition, the study also contributed to the literature about the potential mechanisms of sleep quality as influenced by resilience *via* a mediating model. Based on the theorical framework, we set up perceived stress as the key variable and demonstrated that perceived stress mediated this path among disabled elders in China. Considering the correlations among resilience, sleep quality, and perceived stress indicated by our research, we proposed that, while improving psychological resilience, invention of a program to reduce perceived stress will also be a valid method to increase a person’s quality of sleep, especially among disabled older adults. The disabled elders were a special older population who have impairment of body or spirit. Compared to general elders, they will suffer from more adverse physical and psychological experience. More concern and care should be taken by individuals, governments, and society for this special older population—disabled elders.

## Data Availability Statement

The raw data supporting the conclusions of this article will be made available by the authors, without undue reservation.

## Ethics Statement

The studies involving human participants were reviewed and approved by the Ethics Committee of East China University of Science and Technology. The patients/participants provided their written informed consent to participate in this study.

## Author Contributions

YC and LH designed the study. YC, JW, and LH performed the study and wrote the manuscript together. All authors have read and approved the final version of the manuscript.

## Conflict of Interest

The authors declare that the research was conducted in the absence of any commercial or financial relationships that could be construed as a potential conflict of interest.
